# The “Register”

**Published:** 1898-01

**Authors:** 


### EDITORIAL.

### The "Register."

The present issue is the beginning of the fifty-second year of the REGISTER. It began its career as a quarterly, and a child of the Mississippi Valley Dental Society, October, 1847; it consisted of forty-eight pages. The first editors were Drs. S. P. Hullihen, of Wheeling, Va.; Dr. B. B. Brown, of St. Louis, Mo., and James Taylor, of Cincinnati, O. It was published in the latter city under the immediate supervision of Dr. Taylor. The editorial staff changed from time to time. Dr. Taylor was managing editor for nine years. Two years after its establishment the journal became the property of Dr. Taylor; and was owned and edited by him till the ninth volume when it became the property of Drs. Taft and Watt; and was edited by them till 1872, since which time it has been the property of and edited by J. Taft.

The REGISTER was issued as a quarterly, till the fourteenth volume, when it became a monthly in 1860, the first monthly dental journal in this country; or in the world.

No special claim is made on account of the age of the REGISTER, beyond the fact that it was one of the first journals in the field for the promotion, development and progress of dentistry, and it is to be hoped that its work has not been in vain. The REGISTER does not claim to be *the* representative dental journal of the world. The effort will be, however, as in the past, to make it a vehicle of useful information for the dental profession, and it is earnestly desired that for the fullest realization in this direction the aid of the profession will be liberally bestowed upon it, both financially, and by the contribution of useful and interesting matter for its pages. We would have none write less for other journals, than heretofore, but more for the REGISTER. The value and interest of any journal very largely depends upon the co-operation of the contributors to its pages. Much is written that is not regarded as appropriate for publication in a professional journal, and so does not appear; but let not that fact deter any one from writing. There are many new thoughts, ideas and discoveries that ought to be preserved and made useful in the future. Many in the profession do not even speak of such things, a few do however speak of them to their confreres, either singly or in associations. This is not enough; every valuable thought or discovery should have permanent record on the printed page, where it may serve valuable purpose, not only now, but in the indefinite future.

There will be many in the next generation of dentists who will do more and better in these matters than their fathers have done. So mote it be.

